# Functional sugar-free chewing gum infused with spices bolsters antioxidant capacity and phenolic content of saliva

**DOI:** 10.1038/s41598-023-30931-2

**Published:** 2023-03-23

**Authors:** Kristi M. Crowe-White, Seung Eun Jung, Anna Bragg, Katelyn E. Senkus

**Affiliations:** Department of Human Nutrition, The University of Alabama, 401 Russell Hall, Box 870311, Tuscaloosa, AL 35487 USA

**Keywords:** Cardiovascular diseases, Dental diseases, Prognostic markers

## Abstract

Oral and vascular diseases are seemingly disparate conditions, yet individuals with poor oral health are at increased risk for cardiovascular events. Spice-derived bioactive polyphenols with antioxidant functionality may attenuate mechanisms linking the diseases, namely oxidative stress and inflammation. Acknowledging that novel approaches to increase antioxidant intake are warranted, the purpose of this study was to evaluate the influence of two functional sugar-free gums infused with spices on antioxidant capacity and phenolic content of saliva using the oxygen radical absorbance capacity and Folin-Ciocalteu assays, respectively. Unstimulated followed by stimulated saliva was collected according to a validated method across a prescribed five minute chewing period. Both gums significantly increased hydrophilic, lipophilic, and total antioxidant capacity of saliva (p < 0.05) yet to varying extents. Phenolic content of saliva was significantly higher (p < 0.001) post-chew for both gums. Results suggest spices infused into sugar-free chewing gum bolster the antioxidant capacity of saliva, thereby promoting oral health. Research evaluating the sublingual absorption of spice-derived antioxidants in functional gums and their influence on systemic oxidative stress is warranted.

## Introduction

Periodontitis and cardiovascular disease (CVD) may appear as disparate health conditions, yet longitudinal studies suggest that individuals with poor oral health or periodontal disease are at significantly increased risk for cardiovascular events^1^. Biologically plausible mechanisms linking these diseases are localized oxidative stress and inflammation in the oral cavity with systemic translocation influencing cells of the vascular endothelium^2,3^. As CVD is the leading cause of death for both men and women in the United States, it accounts as a significant contributor to health expenditures^4^. These statistics underscore the need for shifting the paradigm from primarily treatment to prevention of mechanisms underpinning both CVD and periodontal disease. In addition to the aforementioned mechanisms linking the two diseases, poor diet quality also plays a role in the etiology of both periodontal disease and CVD^5,6^. In contrast, high quality dietary patterns rich in antioxidants show promise for decreasing both local and systemic oxidative stress^7–9^.

According to the American Dental Association, chewing sugar-free gum for 20 min after a meal may prevent tooth decay—a dental condition influenced by oral oxidative stress^10,11^. Furthermore, the regular use of sugar-free gum has been associated with improving overall oral health and reducing caries although the exact mechanisms are unknown^12^. Nevertheless, it has been noted that saliva constitutes a first line of defense against free radical-mediated oxidative stress^2^. As such, efforts to bolster salivary antioxidant capacity for quenching free radicals in the oral cavity are warranted. One plausible means to achieve this is infusion of antioxidants into chewing gum—a functional convection vehicle for carrying medicines or food-derived bioactive compounds^13^.

Acknowledging the antioxidant functionality of polyphenols, it has been noted that spices are a concentrated source of polyphenols in the diet^14,15^. For example, cinnamon (*Cinnamomum* species) and nutmeg (*Myristica fragrans*) contain an abundance of polyphenols with antioxidant and anti-inflammatory functionality^16,17^. Thus, the addition of these natural ingredients extends beyond merely boosting taste and flavor to providing substrates for quenching free radical species^18^. As such, the purpose of this randomized, single-blind pilot study was to evaluate functional sugar-free gums infused with spices for improving the antioxidant capacity and phenolic content of saliva.

## Materials and methods

### Study design

Eligibility for this study included men and women ages 19 years or older of any ethnic background recruited from The University of Alabama through the use of fliers and advertising on televisions throughout campus. Participants were excluded if they had a soy or latex allergy as well as the presence of active dental caries or any dental device for which chewing gum is not recommended. The Institutional Review Board at The University of Alabama provided study approval (18-OR-059-ME); furthermore, all participants provided written informed consent prior to study initiation. Funding for this study was provided by the Academy of Nutrition and Dietetics Foundation Award and the McCormick Science Institute.

Data was collected from 54 participants (21.4 ± 5.8 years, 18.8% male, 88.6% white/Caucasian). Participants were randomized to evaluate one of two sugar-free gum formulations on two separate testing visits held one week apart. Prior to testing, participants refrained from eating, drinking, or brushing their teeth one hour prior to each gum testing session. At each testing visit, participants were provided with two 1.8 mL cryovials equipped with saliva collection aids to disperse bubbles (Salimetrics, LLC, State College, PA) and a coded piece of gum to ensure participant blinding to gum formulation. Unstimulated followed by stimulated saliva was collected according to a validated method across a prescribed five minute chewing period^19^. In short, saliva was allowed to accumulate in the floor of the mouth and expectorated at one minute intervals. The unstimulated saliva served as the baseline reference for evaluating biochemical outcomes. The same process of collection was repeated during the stimulated saliva collection period while participants were chewing the respective gum for the purpose of evaluating changes in antioxidant capacity in the oral cavity. Upon collection, vials of unstimulated and stimulated saliva for each gum formulation were stored at − 20 °C until time of analysis.

### Gum composition

The composition of the gums is reflected in Table 1 and US Patent Number 11,304,896^20^. All ingredients were food grade including the gum base which was comprised of chicle. Spices and extracts were obtained from McCormick & Company (Baltimore, MD). Following the incorporation of all ingredients, the final size of each formulated gum was approximately 0.5 inch square cube with a mass of 2.72 g. Individual gum chews were packaged identically albeit with a code designating the formulation—gum 1 (G1) or gum 2 (G2).

**Table 1:** Composition of functional chewing gums.

Ingredients	Percent total mass	
	Gum 1 (%)	Gum 2 (%)
Chicle gum base	28.95	29.30
Non-nutritive sweeteners and flavoring agents	62.01	62.59
Texturizing agents	7.00	7.08
Apple pie spice	1.53	NA
Cinnamon spice	0.51	1.03

*G1* Functional Gum 1 infused with cinnamon and apple pie spice, *G2* Functional Gum 2 infused with cinnamon only, *NA* not applicable.

### Saliva analyses

Antioxidant capacity (hydrophilic and lipophilic) of saliva was measured by the oxygen radical absorbance capacity assay on a FLUOstar Optima plate reader (BMG Labtech, Cary, NC) using a validated method by Prior et al.^21^. The compound 2,2-azobis(2-amidino-propane) dihydrochloride was used as the peroxyl radical generator and Trolox, a water-soluble analogue of vitamin E, was used as the reference antioxidant standard. Results are expressed as μM Trolox equivalents (TE). Total antioxidant capacity is representative of the sum of the hydrophilic and lipophilic antioxidant capacities.

Total phenolic content of saliva was determined using the Folin-Ciocalteu spectrophotometric assay^22^. Briefly, 10% (v/v) Folin-Ciocalteu reagent and 7% (w/v) Na_2_CO_3_ were added to the diluted samples, followed by incubation at 45 °C for 15 min. Samples were read at 765 nm against a gallic acid standard curve. Results are expressed as mg gallic acid equivalents (GAE).

### Statistical analysis

For pre- and post-chew saliva collected for both gum formulations, results were evaluated using paired t-tests. Independent t-tests were used to assess differences between gum formulations. Antioxidant capacity and total phenolic data are provided as mean and standard deviation. A significant threshold of increase in antioxidant capacity was defined as 25%. Thus, power analysis generated a sample size of n = 35 for 80% power at an alpha level of 0.05.

### Ethics approval

Approval was obtained from the ethics committee of The University of Alabama. The procedures used in this study adhere to the tenets of the Declaration of Helsinki.

### Consent to participate and for publication

Per Institutional guidelines, informed consent was obtained from all participants for study participation as well as for use of data in publications.

## Results

**Figure 1:**
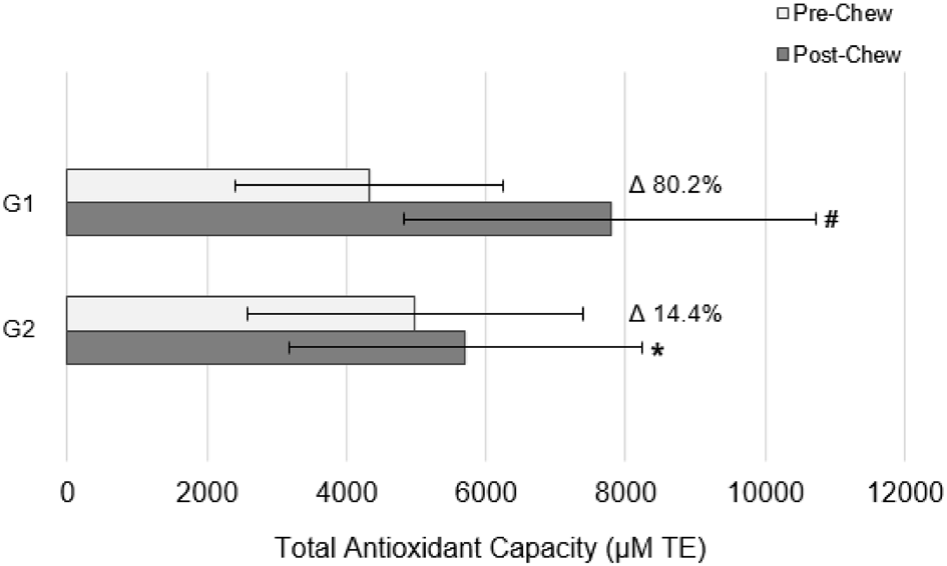
Changes in salivary total antioxidant capacity after chewing functional sugar-free gums infused with spices. *G1* Functional Gum 1 infused with cinnamon and apple pie spice (containing cinnamon and nutmeg), *G2* Functional Gum 2 infused with cinnamon only, *TE* trolox equivalents. *p < 0.05 within group difference; ^#^p < 0.001 within group difference.

Both gums significantly increased hydrophilic, lipophilic, and total antioxidant capacity of saliva (p < 0.05), yet the greatest release of antioxidants resulted from G1 infused with cinnamon and apple pie spice (containing cinnamon and nutmeg) (p < 0.001, all). Chewing G1 resulted in a mean increase in total antioxidant capacity of 80.2% (Fig. 1) (Post-chew: 7845.13 ± 2963.04 μM TE). Accordingly, hydrophilic and lipophilic antioxidant capacity increased by 60.9% and 93.0%, respectively (Hydrophilic—Post-chew: 2800.95 ± 1074.35 μM TE; Lipophilic—Post-chew: 5044.18 ± 2016.05 μM TE). Chewing G2 containing cinnamon only resulted in a mean increase in total antioxidant capacity of 14.4% (Fig. 1**)** (Post-chew: 5708.63 ± 2520.79 μM TE). Similarly, hydrophilic and lipophilic antioxidant capacity increased by 11.2% and 16.1%, respectively (Hydrophilic—Post-chew: 1901.66 ± 851.32 μM TE; Lipophilic—Post-chew: 3806.97 ± 1714.42 μM TE).

**Figure 2:**
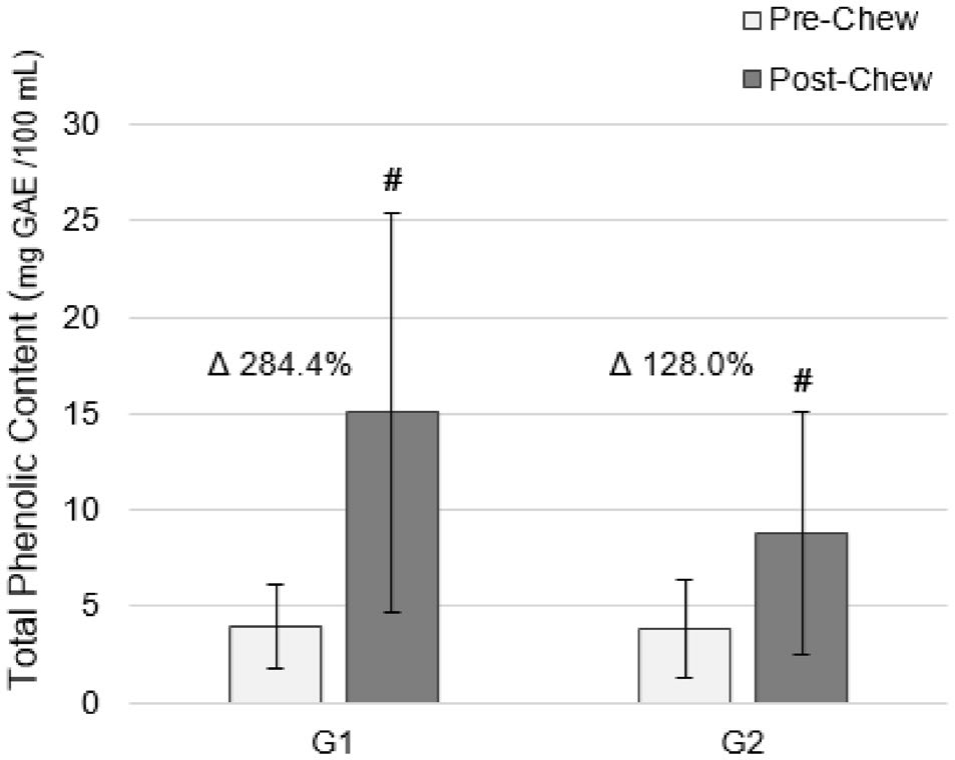
Changes in salivary total phenolic content after chewing functional sugar-free gums infused with spices. *G1* Functional Gum 1 infused with cinnamon and apple pie spice (containing cinnamon and nutmeg), *G2* Functional Gum 2 infused with cinnamon only, *GAE* gallic acid equivalents. ^#^p < 0.001 within group difference.

The total phenolic content of saliva was significantly increased (p < 0.0001) post-chew for both gums (Fig. 2). Compared to G2 infused with cinnamon only, a significantly greater increase (p < 0.001) was observed after chewing G1 infused with cinnamon and apple pie spice (containing cinnamon and nutmeg) such that salivary phenolic content increased by 284.4% (Post-chew: 14.99 ± 10.31 mg GAE). Chewing G2 resulted in a 128.0% increase in salivary phenolic content (Post-chew: 8.80 ± 6.33 mg GAE).

**Figure 3:**
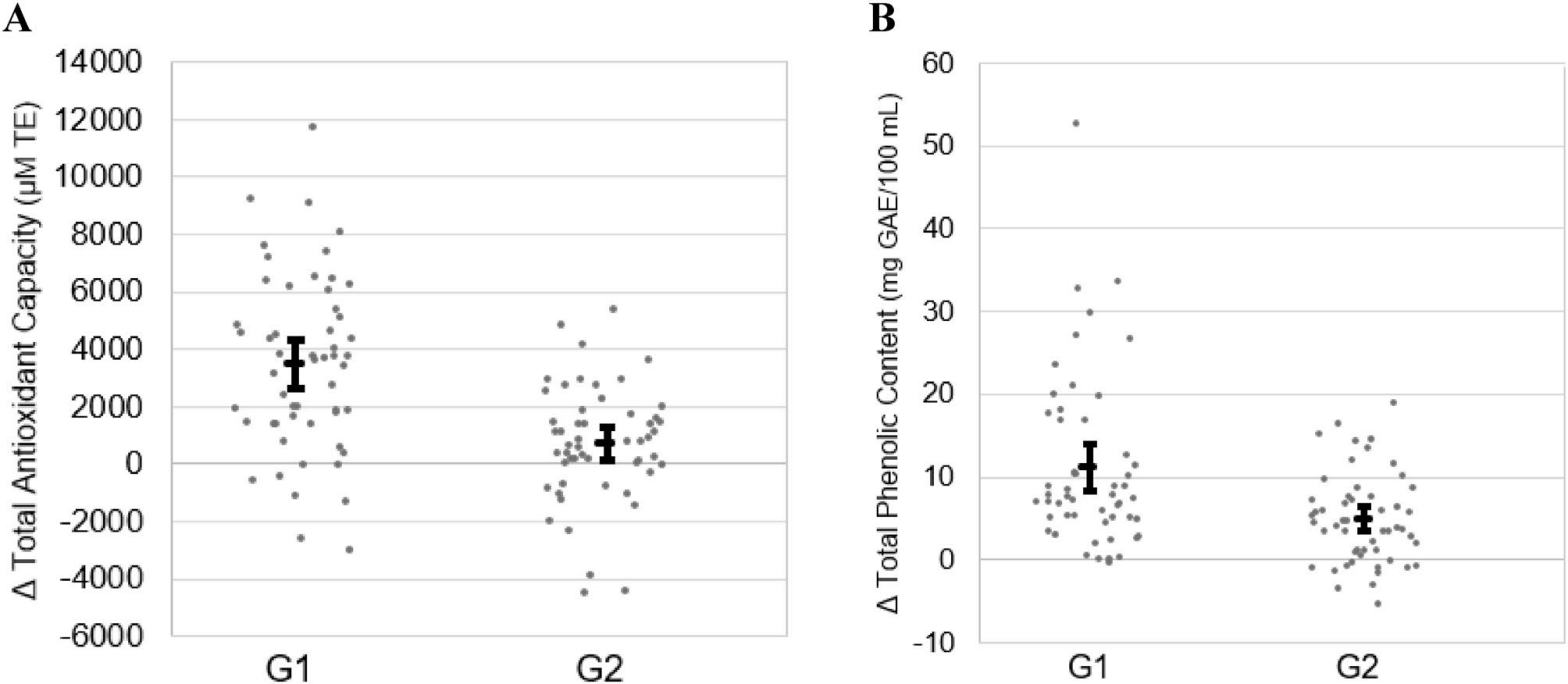
Individual changes in (**A**) salivary total antioxidant capacity and (**B**) salivary total phenolic content after chewing functional sugar-free gums infused with spices. Results represented as individual change values as well as mean and 95% confidence interval. *G1* Functional Gum 1 infused with cinnamon and apple pie spice (containing cinnamon and nutmeg), *G2* Functional Gum 2 infused with cinnamon only, *TE* trolox equivalents, *GAE* gallic acid equivalents.

While there was an overall increase in salivary total antioxidant capacity and total phenolic content for each functional gum, considerable inter-individual differences in response were observed (Fig. 3). For G1 containing cinnamon and apple pie spice, changes in salivary total antioxidant capacity ranged from − 2942.4 to 11,786.0 μM TE and changes in total phenolic content ranged from − 0.2 to 52.8 mg GAE. Six participants experienced a reduction in salivary total antioxidant capacity; whereas, total phenolic content was reduced in only one participant. For G2 (cinnamon only), changes in salivary total antioxidant capacity ranged from − 4470.9 to 5415.2 μM TE and changes in total phenolic content ranged from − 5.21 to 18.95 mg GAE. For this second gum formulation, reductions in salivary total antioxidant capacity and total phenolic content were observed in 13 and 12 participants, respectively.

## Discussion

The purpose of this randomized, single blind pilot study was to evaluate functional sugar-free gums infused with spices for improving antioxidant capacity and phenolic content of saliva. Results reveal improvements resulting from both functional gums, yet G1 comprised of cinnamon and apple pie spice was more effective than G2 containing only cinnamon at improving salivary outcomes of interest. Inter-individual variability in antioxidant capacity and phenolic content of saliva was noted among participants; however, this is not unexpected given that some saliva is swallowed during gum chewing resulting in a diminished capture of saliva containing bioactive phenolic compounds. Additionally, the frequency of expectoration of saliva was encouraged at one-minute intervals but, ultimately, participants expectorated as needed during saliva collection. Taken collectively, this study further supports the growing investigation into the use of chewing gum for delivering bioactive compounds and influencing other outcomes beyond breath freshening^13,23,24^.

With Americans not meeting the recommended intake of antioxidant-dense foods, novel approaches to boost antioxidant intake are warranted^25,26^. Furthermore, acknowledging the demographic targeted in this intervention, evidence suggests that young adults exhibit many unhealthful eating practices which can increase the risk for CVD^27^. Additionally, more than half of all young adults have at least one CVD risk factor^28^. Thus, this demographic represents an ideal population for targeting a prevention-based intervention rich in antioxidants for combating oxidative stress-mediated conditions namely oral diseases and CVD. Whether targeted to this demographic or the broader community of individuals who chew gum and/or are wishing to improve antioxidant intake, functional chewing gums containing spices represent a feasible option for improving antioxidant intake without necessitating a major lifestyle change. Strengths of this study include its robust use of biochemical testing to assess not only the release of phenolic compounds but also their bioactivity as antioxidants. Nevertheless, the findings are not without limitation as results cannot be extrapolated to systemic circulation, thus warranting future investigation of sublingual absorption of spice-derived antioxidants in functional gums.

## Conclusion

This study is the first of its kind to infuse spices into sugar-free chewing gum for bolstering the antioxidant capacity of saliva thereby providing substrates with the potential to lower local oxidative stress in the oral cavity. Despite the inter-individual biological variability among participants in salivary antioxidant capacity, findings of this study aid in the understandiong of the role of chewing gum to deliver bioactive compounds for health and wellness^13^. Given the flux of saliva with plasma at the mucosal interface, it is plausible that these antioxidant compounds are transmitted into circulation to beneficially influence systemic oxidative and inflammatory stress. As such, additional research is needed to determine dose-duration effects of functional gums on improved antioxidant capacity of saliva as well as systemic impacts on oxidative stress reduction.

## Data Availability

The data that support the findings of this study are available on request from the corresponding author [KMCW].

## Abbreviations

CVD: Cardiovascular disease
G1: Gum formulation infused with cinnamon and apple pie spice
G2: Gum formulation infused with cinnamon only
GAE: Gallic acid equivalents
TE: Trolox equivalents
